# Curcumin Inhibits the Primary Nucleation of Amyloid-Beta Peptide: A Molecular Dynamics Study

**DOI:** 10.3390/biom10091323

**Published:** 2020-09-15

**Authors:** Irini Doytchinova, Mariyana Atanasova, Evdokiya Salamanova, Stefan Ivanov, Ivan Dimitrov

**Affiliations:** 1 Faculty of Pharmacy, Medical University of Sofia, 2 Dunav st., 1000 Sofia, Bulgaria; matanasova@pharmfac.mu-sofia.bg (M.A.); esalamanova@bio21.bas.bg (E.S.); ivanovs@umd.edu (S.I.); idimitrov@pharmfac.mu-sofia.bg (I.D.); 2 Institute for Bioscience and Biotechnology Research, University of Maryland, Rockville, MD 20850, USA

**Keywords:** amyloid-beta peptide, curcumin, ferulic acid, molecular dynamics

## Abstract

The amyloid plaques are a key hallmark of neurodegenerative diseases such as Alzheimer’s disease and Parkinson’s disease. Amyloidogenesis is a complex long-lasting multiphase process starting with the formation of nuclei of amyloid peptides: a process assigned as a primary nucleation. Curcumin (CU) is a well-known inhibitor of the aggregation of amyloid-beta (Aβ) peptides. Even more, CU is able to disintegrate preformed Aβ firbils and amyloid plaques. Here, we simulate by molecular dynamics the primary nucleation process of 12 Aβ peptides and investigate the effects of CU on the process. We found that CU molecules intercalate among the Aβ chains and bind tightly to them by hydrogen bonds, hydrophobic, π–π, and cation–π interactions. In the presence of CU, the Aβ peptides form a primary nucleus of a bigger size. The peptide chains in the nucleus become less flexible and more disordered, and the number of non-native contacts and hydrogen bonds between them decreases. For comparison, the effects of the weaker Aβ inhibitor ferulic acid (FA) on the primary nucleation are also examined. Our study is in good agreement with the observation that taken regularly, CU is able to prevent or at least delay the onset of neurodegenerative disorders.

## 1. Introduction

Amyloidogenic peptides and proteins are soluble structures that are able to aggregate into insoluble amyloid fibrils with a characteristic core structure rich in β-sheets [1]. Evolutionary, these proteins have appeared in the microorganisms as necessary components of physiological functions such as biofilm formation [2], cell adhesion [3], interactions with host membranes [4], and permeabilization [5]. In humans, some of them also show physiological functions. For example, amylin is involved in the regulation of insulin and glucagon secretion [6], tau protein stabilizes the microtubules in the cells [7], the amyloid-beta peptide controls the synaptic activity [8] and acts as a protective agent against brain infections [9,10], α-synuclein regulates the neurotransmitter release [11], and premalanosome protein (PMEL) forms fibrils in the melanocytes required for the optimal cell function [12]. At the same time, the ability of amyloidogenic proteins to form fibrils is a key feature of more than 50 diseases such as Alzheimer’s disease [13], Parkinson’s disease [14], prion disease [15], type 2 diabetes [16], and sickle-cell anemia [17]. The triggers that convert the functional amyloids into pathological ones still remain unclear.

The amyloid fibril formation is a complex process consisting of three phases: nucleation, elongation, and saturation [18]. The nucleation starts with the aggregation of two monomers into a dimer, which further attracts other monomers and evolves into an oligomer. The oligomer might arrange into toxic fibrillar aggregate (on-pathway) or into non-toxic disordered aggregate (off-pathway). The toxic fibrils elongate by attaching novel monomers in an appropriate cross-β-conformation. In the saturation phase, the fibril formation reaches equilibrium as monomers detach and attach continuously at the ends of the fibril. The nucleation could be primary or secondary. A primary nucleation is assigned to the creation of a folding nucleus, from which oligomeric and fibrillar assemblies emanate [19], while the secondary nucleation describes the formation of nuclei around pre-existing crystals [20].

As the nucleation phase is the initial step in the process of fibril formation, inhibition of the aggregation of amyloidogenic peptides and proteins is one of the therapeutic strategies in the development of anti-amyloid drug candidates [21]. It was reported that curcumin (CU), rosmarinic acid, nordihydroquaiaretic acid, rifampicin, and wine-related polyphenols, such as myricetin, dose-dependently inhibit amyloid-beta (Aβ) fibril formation from Aβ_1-40_ and Aβ_1-42_ and destabilize preformed Aβ fibrils in vitro [22].

CU, a polyphenol from Curcuma longa (turmeric plant), is known to treat many diseases, including neurodegenerative ones such as Alzheimer’s disease, Parkinson’s disease, prion disease, multiple sclerosis, stroke, anxiety, and depression [23]. It has been observed that the death rate of Alzheimer’s/dementia per 100,000 population standardized by age in India is significantly lower than in the Middle East [24]. One of the reasons could be the regular intake of turmeric in the routine diet of Indians [25]. CU is a widely studied compound, and many good reviews on its properties and applications are available in the literature [23,26,27,28,29,30]. It was found in vitro that CU blocks the toxicity of Aβ oligomers (IC_50_ = 0.8 µM) [31] and disintegrates preformed Aβ fibrils (EC_50_ = 1 µM) [32]. In animals, it was shown that CU reduces insoluble Aβ deposits [33] and amyloid plaques [31]. CU formulations with improved bioavailability have been demonstrated to improve memory, mood, and alertness in clinical trials with non-demented elderly patients [34,35].

The interactions of CU with Aβ peptides and fibrils have been simulated by molecular dynamics (MD). Zhao et al. have studied the effects of CU on the stability of Aβ dimers and have found that CU acts as β-sheet breaker, reducing the β-sheet content within the Aβ oligomers [36]. Awasthi et al. have analyzed the effects of CU on the stability of two familial Aβ42 mutations—A2V (harmful) and A2T (protective)—and have found that the mutant A2V is more stable than A2T in the absence of CU, while A2T is more stable in the presence of CU [37]. Apart from oligomers, CU binds tightly to preform Aβ fibrils occupying the binding pocket inside fibrils [38]. It forms hydrogen bonds and hydrophobic interactions with protofibrils [38,39] and causes structural distortion and perturbation [39,40], leading to the dissociation of the outermost peptides [41]. The number of intermolecular backbone hydrogen bonds between adjacent β-threats in fibrils correlates with the anti-aggregation activity of Aβ inhibitors [42].

The aim of the present study was to simulate by MD the primary nucleation of an ensemble of 12 Aβ_1-42_ peptides and to analyze the effects of the inhibitor curcumin. For comparison, the effects of the weaker inhibitor ferulic acid (FA) (IC_50_ = 5.5 µM) [43] were also conducted.

## 2. Models and Method

### 2.1. Modeled Ligands and Systems

**Figure 1:**
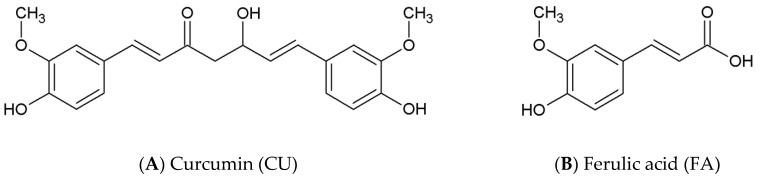
Chemical structures of the amyloid-beta (Aβ) inhibitor (**A**) curcumin (CU) and (**B**). non-inhibitor ferulic acid (FA).

The chemical structures of CU (CID 969516) and FA (CID 445858) were retrieved from PubChem [44] (Figure 1). The keto-enol form of CU was used in the present study instead of β-diketone, as it has been proven that the keto-enol form exhibits anti-aggregatation properties and is responsible for the ability of CU to prevent Aβ oligomer and fibril assembly [45,46]. FA is a weak acid with pK_a_ 4.58 and at physiological pH 7.4, the anionic form dominates. This form was used in the simulations. The molecules were parameterized by GAFF 2.11 [47] with AM1–BCC charges [48] before being used in Amber 18 [49]. The Aβ_1-42_ monomer structure was obtained from PDB (Protein Database); (pdb code 1IYT) [50]. Both ends were capped, and all hydrogens were added.

**Figure 2:**
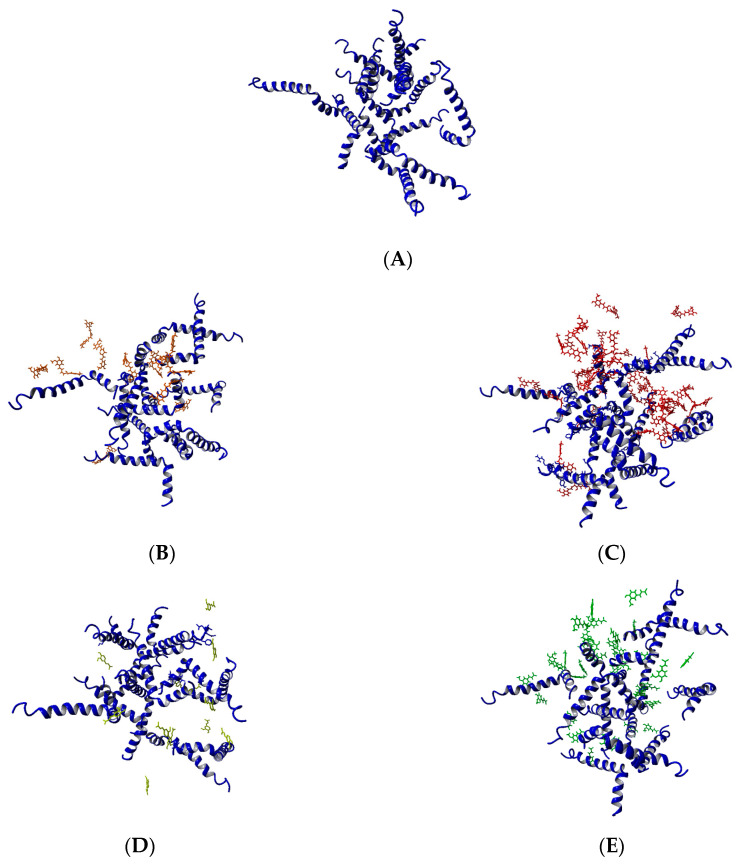
Modeled systems in the present study: (**A**) 12 Aβ monomers; (**B**) 12 Aβ monomers and 12 CU molecules; (**C**) 12 Aβ monomers and 36 CU molecules; (**D**) 12 Aβ monomers and 12 FA anions; (**E**) 12 Aβ monomers and 36 FA anions.

Five systems were modeled (Figure 2). The first one contained 12 Aβ_1-42_ monomers randomly positioned in a truncated octahedron cell with a volume of 1,927,025 Å^3^ and solvated with TIP3P water yielding 10.4 mM solution. NaCl was added to maintain a physiological salt concentration and adjusted to neutralize the peptides’ charge. This system was used for the construction of the other four systems. Two of them contained 12 and 36 CU molecules spread randomly around the Aβ monomers corresponding to 9.3 mM and 30.4 mM solutions, respectively. The last two systems included 12 and 36 FA anions placed around the Aβ monomers, giving 10.3 mM and 31.2 mM solutions, respectively. The salt concentration was adjusted additionally to neutralize the FA charge.

### 2.2. MD Protocol

Initially, the solvated systems were energy minimized for 5000 steps with 3 kcal/molÅ^2^ harmonic restraints on solute heavy atoms, followed by heating them from 0 to 300 K over a period of 1 ns at constant volume with identical restraints. Next, the systems were equilibrated for 1 ns of constant pressure density with restraints and for 100 ns without any restraints. Finally, they were simulated for 1000 ns of production dynamics under constant temperature (310 K) and pressure (1 bar), maintained with the Langevin thermostat [51] and Berendsen barostat [52], respectively. The systems were simulated with the ff14SB [53] force field under periodic boundary conditions. A 12.0 Å cutoff was used for both van der Waals and electrostatic interactions; long-range electrostatics beyond the real-space cutoff were evaluated with the particle-mesh Ewald (PME) scheme [54]. During heating, density equilibration, preproduction, and production dynamics, covalent bonds involving hydrogen were constrained using the SHAKE algorithm [55], allowing a 2 fs time step; only during energy minimization were the bonds to hydrogen were not constrained. During production dynamics, frames were saved every 1 ns for a total of 1000 per trajectory to be used in subsequent analysis. The MD simulations were performed by Amber 18 [56] and were analyzed by cpptraj V4.24.0 [57].

## 3. Results

The five modeled Aβ ensembles—12 Aβ_1-42_ monomers (10.4 mM), 12 Aβ_1-42_ monomers + 12 CU molecules (9.3 mM), 12 Aβ_1-42_ monomers + 36 CU molecules (30.4 mM), 12 Aβ_1-42_ monomers + 12 FA anions (10.3 mM), and 12 Aβ_1-42_ monomers + 36 FA anions (31.2 mM) (Figure 2)—were simulated in saliva for 1 µs according to the MD protocol described in Models and Methods. The modeled Aβ ensembles formed stable nuclei for a different period of time. The coordinate trajectories were processed and used to analyze the effects of CU and FA on the process of primary nucleation of Aβ peptides.

### 3.1. Curcumin Stabilizes Immediately the Aβ_1-42_ Ensemble

The averaged backbone RMSDs (root mean square deviations) of the ensembles of 12 Aβ_1-42_ peptides with and without ligands were calculated as a function of time (Figure S1). The ensemble without a ligand underwent conformational distortions during the initial 300 frames (300 ns) and then was stabilized (Figure S1A). The presence of a ligand stabilizes the Aβ ensemble earlier. In the presence of 12 CU molecules, the ensemble was stabilized for 8 ns (Figure S1C); in the presence of 36 CU, it was stabilized for 2 ns (Figure S1E); in the presence of 12 FA, it was stabilized for 20 ns (Figure S1G); and in the presence of 36 FA, it was stabilized for 15 ns (Figure S1I). It is clearly evident that the presence of CU molecules stabilizes immediately the ensemble in a dose-dependent manner. Two additional 50 ns productions on the five systems were run. They confirmed the immediate stabilizing effect of CU (Figure S2).

**Figure 3:**
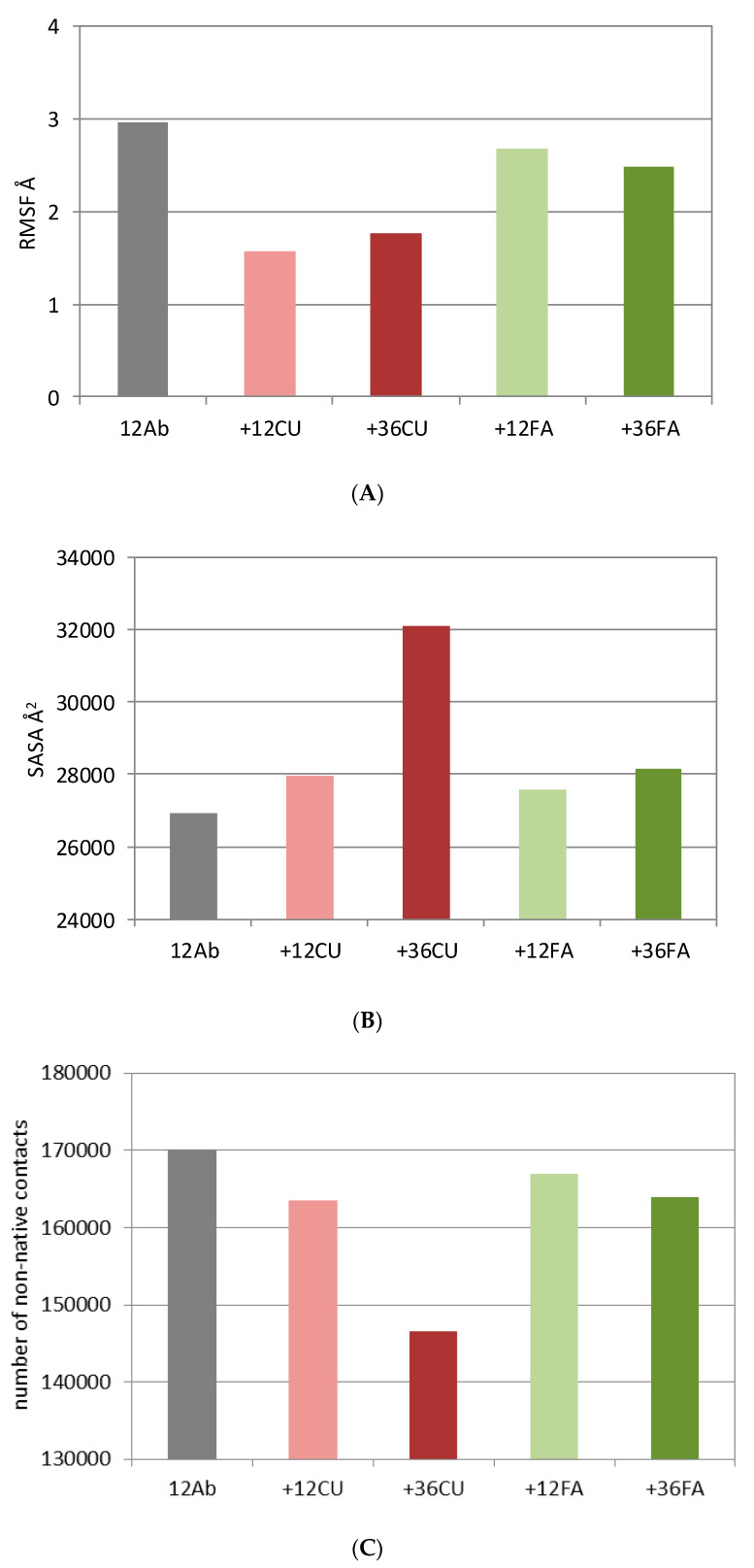

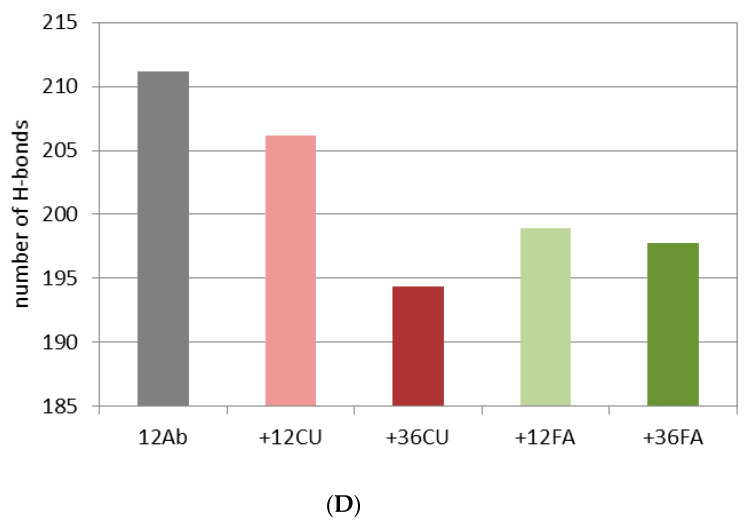
Root mean square fluctuations (RMSF) (**A**), solvent-accessible surface area (SASA) (**B**), number of non-native contacts (**C**), and number of H-bonds (**D**) averaged over 1000 frames (1000 ns) for 12 Aβ monomers (gray), 12 Aβ monomers and 12 CU molecules (pink), 12 Aβ monomers and 36 CU molecules (red), 12 Aβ monomers and 12 FA anions (light green), 12 Aβ monomers and 36 FA anions (dark green).

The backbone RMSFs (root mean square fluctuations) indicate the most fluctuating Aβ residues during the MD simulation. Typically, the terminals of the Aβ peptides fluctuate more than the middle parts, with the N-terminals being the most flexible. The averaged RMSFs of the ensembles of 12 Aβ peptides with and without ligands are summarized in Figure 3A. The presence of any ligand reduces the fluctuations of the Aβ ensemble. The effects of CU are more prominent than the effects of FA. CU decreases mainly the fluctuations of the C-terminals (Figure S1D,F).

### 3.2. Curcumin Binds inside the Aβ Nucleus

**Figure 4:**
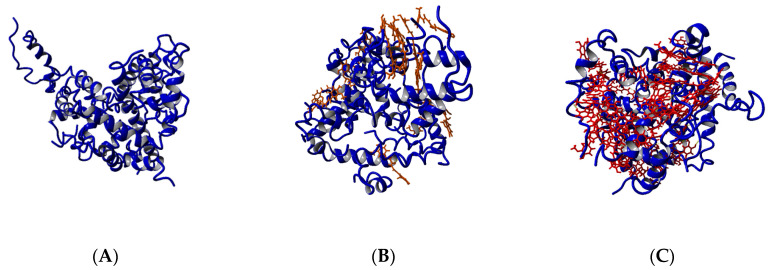

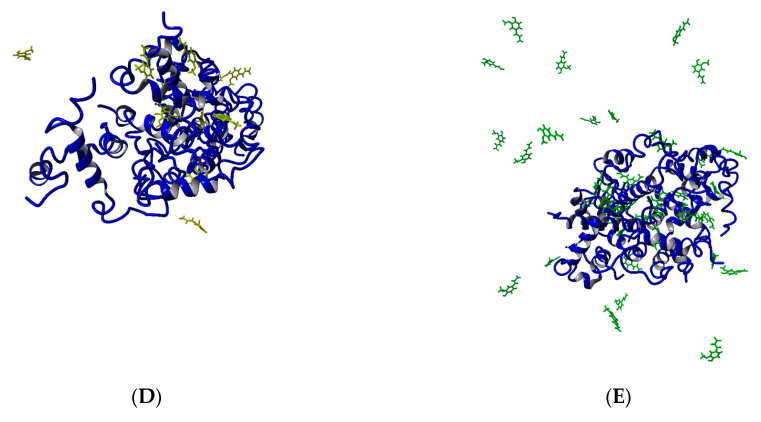
The modeled systems after 1 µs molecular dynamics (MD) simulation: (**A**) 12 Aβ monomers; (**B**) 12 Aβ monomers and 12 CU molecules; (**C**) 12 Aβ monomers and 36 CU molecules; (**D**) 12 Aβ monomers and 12 FA anions; (**E**) 12 Aβ monomers and 36 FA anions.

The coordinates of the modeled systems after 1 µs MD simulations are given in Figure 4. In 300 ns, the 12 Aβ monomers collapsed into a compact nucleus losing part of the ordered structures (α-helix, 3-10 helix, β-turn) (Figure S2). The Aβ_1-42_ peptide is a water-soluble molecule containing 20 hydrophobic residues (Ala, Ile, Leu, Met, Val, Phe, Tyr, and Trp) clustered mainly in the middle part of the molecule and in the C-terminal. The Aβ_1-42_ peptide has an overall logP −12.2 [44] and isoelectric point pI 5.5 [58]. In polar medium, the flexible peptide chains adopt spontaneously a globular conformation with a polar surface and non-polar core (Figure 4A). As a typical hydrophobic interaction, the nucleation is an entropy-driven process governed by an increase of solvent entropy.

Regardless of the concentrations, all molecules of the strong inhibitor CU were bound to Aβ peptides (Figure 4B,C). Some of them were intercalated and bound inside the core, while others covered the surface. The main interactions between CU and Aβ residues were hydrophobic, but π–π interactions with the side chains of Phe4, Tyr10, Phe19, and Phe20 and cation–π interactions with the side chains of Arg5, Lys16, and Lys28 also occurred. Plenty of H-bonds exist between CU molecules and Aβ residues. The hydrogen bonds in the ensembles are considered in detail in Section 3.6.

The Aβ ensemble has quite a different look in the presence of the weak inhibitor FA (Figure 4D,E). In the presence of 12 FA, after 1 µs simulation, nine of them (75%) were bound to Aβ peptides, while in the presence of 36 FA, this number was only 18 (50%). Here again, the main interactions between the FA molecules and Aβ peptides were hydrophobic. Most of the bound molecules made π–π stacking with the aromatic side chains of Phe4, Tyr10, Phe19, and Phe20. Cation–π interactions appeared between the phenyl ring of FA and the side chains of Arg5, Lys16, and Lys28. The hydrogen bonds between Aβ and FA anions are considered in detail in Section 3.6.

Both ligands disrupted the salt bridges formed between the Aβ chains. Their number decreases from 142 (without ligands) to 121 (with 12 CU), 119 (with 36 CU), 123 (with 12 FA), and 118 (with 36 FA). The salt bridges were formed between Asp1, Asp7, Asp23, Glu3, Glu22, Arg5, Lys16, and Lys28.

### 3.3. Curcumin Does Not Affect the Secondary Structures of the Peptides in the Aβ_1-42_ Ensemble

The analysis of the secondary structures of the Aβ peptides indicates that the ensembles with and without ligands underwent similar changes during the simulations. The total structural propensities over the initial and final 100 ns are given in Figure S3. The propensities of helices (including 3–10 helices) decreased by 8% (12 Aβ) to 18% (12 Aβ + 12 FA). The β-turn propensities decreased for the ensembles of 12 Aβ peptides, 12 Aβ + 12 CU, and 12 Aβ + 36 FA, for the ensemble of 12 Aβ + 12 FA, they increased, and the ensemble of 12 Aβ + 36 CU remained almost unchanged. The bending propensities in all ensembles increased by 13% (12 Aβ + 36 FA) to 23% (12 Aβ + 36 CU). Indeed, the nucleation proceeds by bending and losing the helical and β-turn structures (Figure 4). The final ensembles are nuclei of randomly folded chains. A distinguishable effect of CU on the secondary structures of Aβ peptides during the 1 µs simulation of the process of nucleation was not observed.

### 3.4. Curcumin Increases the Solvent-Accessible Surface Area (SASA) of the Aβ_1-42_ Ensemble

The solvent-accessible surface area (SASA) was calculated using the algorithm of Weiser et al. [59] and expressed in Å^2^. The SASAs of the five ensembles averaged over 1000 frames are given in Figure 3B. CU at higher concentration (36 CU) increases the SASA of the 12 Aβ ensemble by 20%. At lower concentration (12 CU), the increase is only 4% comparable to the increase caused by 12 FA and 36 FA. The increased surface area is associated by the many CU molecules bound among the folded Aβ chains (Figure 4).

### 3.5. Curcumin Decreases the Number of Non-Native Contacts in the Aβ_1-42_ Ensemble

Amber defines as a non-native contact any contact between a pair of atoms satisfying the distance cutoff (by default 7 Å), which is not already a native contact [56]. The number of the non-native contacts between the Aβ monomers in the five ensembles averaged over 1000 frames are given at Figure 3C. The CU molecules at a higher concentration decrease the number of the non-native contacts in the Aβ ensemble by 16%, while the decrease caused by other ligands varied between 2% for 12 FA and 4% for 12 CU and 36 FA. The reduced contacts between the Aβ monomers are due to the intercalation of many CU molecules bound inside the Aβ nucleus (Figure 4).

### 3.6. Curcumin Decreases the Number of Hydrogen Bonds in the Aβ_1-42_ Ensemble

The number of hydrogen bonds averaged over 1000 frames of the five Aβ ensembles are plotted at Figure 3D. Here again, CU at higher concentration decreases the number of H-bonds the most. The CU molecules bound internally disrupt the pre-existing H-bonds between the Aβ monomers and/or prevent the formation of new ones (Figure 4B,C). As expected, FA as an anion and H-bond acceptor also decreases the number of H-bonds by replacing the peptide residues in some of them (Figure 4D,E).

In total, between 2191 and 2840 H-bonds were formed in the ensembles during the 1 µs MD simulation (Table 1). Of them, 73–90% were between the Aβ chains, 10–26% were between Aβ and ligands, and 0.15–4% were between ligands. In the presence of 12 and 36 CU molecules, the average lifetimes of the H-bonds between the Aβ peptides were 41.7 ns and 36.4 ns, respectively, 39.05 ns on average. The corresponding lifetimes for FA anions were 33.9 ns and 38 ns, on average 35.95 ns. The lifetimes of the H-bonds between Aβ peptides and ligands were on average 14.55 ns for CU and 5.7 ns for FA. The H-bonds formed between ligands lived on average 9.8 ns for CU and 2.05 ns for FA. It is clearly evident that CU decreases the number of H-bonds between Aβs without affecting their lifetimes, and it stays bound longer to Aβ chains and to other CU molecules than FA anions do. The lifetimes of H-bonds in the Aβ ensembles are given in detail in Figure S4.

In 71% of the H-bonds with Aβ peptides, CU acts as an H-bond donor (Table 1) through the phenolic OH groups and the enol group, which interacts with the side chains of Asp1, Arg5, Asp7, Asp23 and the backbone carbonyl oxygens of Gly9, Lys28, and Leu34. In 29% of the H-bonds, CU is a H-bond acceptor through the keto O-atom such as in the bond with the guanidino group of Arg5 or in the bond with the backbone N-atom of Lys28. In contrast, the FA acts mainly as an H-bond acceptor (82% in average) through the phenolic and carboxy O-atoms.

## 4. Discussion

In the present study, the primary nucleation of 12 Aβ_1-42_ peptides was modeled by molecular dynamics in saline at 37 °C for 1 μs. CU is a well-known inhibitor of Aβ self-assembly [58]. At pH 7.4, it is a neutral hydrophobic molecule that is slightly soluble in water (Table 2). The enol form of CU has 11 rotatable bonds, 3 HB donors, and 6 HB acceptors. CU has poor bioavailability (up to 1%) [59] requiring high doses (from 2 to 12 g) to achieve therapeutic concentrations [60]. It crosses the blood–brain barrier (BBB), binds to Aβ peptides, and blocks the aggregation and fibril formation in vitro and in vivo [31]. Even more, CU increases the phagocytosis of Aβ peptides, leading to the effective clearance of plaques from the brain of patients with Alzheimer’s disease [31]. As a diferuloylmethane, CU could be considered as consisting of two FA molecules. FA is the most abundant phenolic acid in whole grain wheat [61,62]. It has strong antioxidant capacity [63,64] and positively affects inflammation, diabetes, cancer, aging, and neurodegeneration [65,66,67]. FA is a weak acid that is freely soluble in water with four rotatable bonds, two HB donors, and four HB acceptors (Table 2). At pH 7.4, the anionic form dominates. As in CU, FA has poor bioavailability [68] but crosses the BBB [69]. Reinke et al. [70] have found that two aromatic rinds are required for the inhibition of Aβ aggregation, and the optimal linker between them should be rigid (up to two rotatable bonds) with a length between 8 and 16 Å. Accordingly, the linker in the enol form of CU consists of seven carbon atoms; five of them are in the sp^2^ state and two are in sp^3^, corresponding to two rotatable bonds. The linker length is 12 Å. FA contains only one aromatic ring and is a weaker inhibitor of Aβ aggregation than CU.

It was found that the IC_50_ values for the inhibition of Aβ aggregation are 0.8 µM for CU and 5.5 µM for FA (Table 2). In the present study, CU was used as a referent inhibitor of Aβ nucleation, while FA was used as a referent weak inhibitor, and the effects of both compounds on the primary nucleation of Aβ_1-42_ peptides were studied. As the process of primary nucleation takes several days in vitro and decades in vivo [73], in order to accelerate the in silico simulation, the systems in the present study were modeled to contain molecules in high concentrations: from 9.3 to 31.2 mM. In 10.4 mM solution of Aβ peptides, the nucleation proceeded in 300 ns. A compact nucleus was formed with a micelle-like architecture. A similar, micelle-like architecture with a hydrophobic core and polar surface was observed in the Monte Carlo simulations of Aβ_1-40_ and Aβ_1-42_ monomers [74]. Based on NMR analysis, Roche et al. [75] found that the self-association of Aβ_1-42_ peptides into toxic oligomers is driven by intermolecular interactions between the hydrophobic regions of the peptides instead of elevated propensities of the monomeric species to adopt β-strand-like conformations.

The addition of ligands in ratios 1:1 and 1:3 accelerated the process dose-dependently. However, the cores that were formed were bigger with up to a 20% increase in SASA. In them, the Aβ chains were less flexible and more disordered. In addition, the ligands decreased the number of non-native contacts and hydrogen bonds between the peptides. Interestingly, the presence of ligands in the ensembles did not affect the longevity of the H-bonds between the peptide chains. More than half of them were short-living (less than 10 ns), one third lived between 10 and 100 ns, and only 12% were long-living (more than 100 ns).

CU binds tightly to Aβ peptides by a wide variety of intermolecular interactions: hydrogen bonds, hydrophobic interactions, π–π stacking, and cation–π attraction. Up to 20% of the hydrogen bonds in the ensembles were formed between the CU molecules and the Aβ peptides. Although CU contains more HB acceptors than HB donors, in 71% of the HB, it acted as a donor. The average lifetimes of the CU–Aβ H-bonds were between 14.3 and 14.8 ns. The longest-living HBs (454–506 ns) were with Asp1. CU made π–π interactions with the aromatic residues in Aβ (Phe4, Tyr10, Phe19, and Phe20) and cation–π interactions with the cationic residues (Arg5, Lys16, and Lys28).

FA binds loosely to Aβ peptides. At the end of the 1 µs simulation, between 25% and 50% of the FA molecules were flanking freely around the peptides. The rest were bound to Aβ by short-living H-bonds (lifetimes between 4.2 and 7.2 ns), hydrophobic interactions, π–π stacking with the aromatic residues, and cation–π interactions with the cationic residues.

## 5. Conclusions

In conclusion, both ligands CU and FA affect the process of primary nucleation of Aβ1-42 peptides, but the effects of CU are more prominent. The CU molecules intercalate among the Aβ chains and bind tightly to them by H-bonds, hydrophobic, π–π, and cation–π interactions. In the presence of CU, the primary nucleus is bigger, while the Aβ peptides are less flexible and more disordered, making fewer non-native contacts and H-bonds. The observed interactions act synergistically and explain at an atomistic level the better inhibitory activity of CU on Aβ aggregation than that of FA. Taken regularly, CU is able to inhibit the process of primary nucleation of Aβ peptides and thus to prevent or at least to delay the onset of neurodegenerative disorders such as Alzheimer’s disease.

## Figures and Tables

**Table 1:** Number, percentage, and lifetime of hydrogen bonds formed between Aβ peptides, between Aβ peptides and ligands, and between ligands calculated over 1000 frames (1000 ns).

Hydrogen Bonds	12 Aβ	12 Aβ + 12 CU	12 Aβ + 36 CU	12 Aβ + 12 FA	12 Aβ + 36 FA
Total	2191	2304	2692	2632	2840
Between Aβ peptides	2191 (100%)	2010 (87%)	2041 (76%)	2356 (90%)	2074 (73%)
Average lifetime (ns)	38	41.7	36.4	33.9	38
Between Aβ and ligands	-	277 (12%)	545 (20%)	272 (10%)	738 (26%)
Average lifetime (ns)	-	14.3	14.8	7.2	4.2
Ligand is a donor	-	197 (71%)	386 (71%)	46 (17%)	139 (19%)
Ligand is an acceptor	-	80 (29%)	159 (29%)	226 (83%)	599 (81%)
Between ligands	-	17 (1%)	106 (4%)	4 (0.15%)	28 (1%)
Average lifetime (ns)	-	8.3	11.3	2.5	1.6

**Table 2:** Physicochemical and ADME (adsorption, distribution, metabolism and excretion) properties of curcumin (CU) and ferulic acid (FA).

Property	Curcumin (CU)	Ferulic Acid (FA)
Molecular weight (g/mol) ^a^	370.38	194.18
Solublity (mg/L) ^b^	3.12	780
LogP ^a^	2.52	1.64
pK_a_^a^	9.73; 10.02; 13.3	4.04; 10.22
Polar surface area (Å) ^a^	96.22	66.76
Free rotatable bonds ^a^	11	4
H-bond donors ^a^	3	2
H-bond acceptors ^a^	6	4
IC_50_ µM (inhibition of Aβ_1-42_ aggregation)	0.8 ^c^	5.5 ^d^
Bioavailability % ^e^	0.47–1	1
Half-life (h)	12.85 ^f^	1 ^g^
BBB permeability	Yes ^f^	Yes ^g^

^a^ Calculated by ACD/Labs (ACD/ChemSketch, version 2020.1.0); ^b^ PubChem [44]; ^c^ [31]; ^d^ [43]; ^e^ DrugBank [71]; ^f^ [68]; ^g^ [72].

## Supplementary Materials

The following are available online at https://www.mdpi.com/2218-273X/10/9/1323/s1. Figure S1. RMSDs (left graphs) and RMSFs per residue (right graphs) averaged over 1000 frames (1000 ns) for of 12 Aβ monomers (grey), 12 Aβ monomers and 12 CU molecules (pink), 12 Aβ monomers and 36 CU molecules (red), 12 Aβ monomers and 12 FA anions (light green), 12 Aβ monomers and 36 FA anions (dark green). Figure S2. RMSDs (˗˗˗single run, ----average of 3 runs) averaged over 50 frames (50 ns) for 12 Aβ monomers (grey, A), 12 Aβ monomers and 12 CU molecules (pink, B), 12 Aβ monomers and 36 CU molecules (red, C), 12 Aβ monomers and 12 FA anions (light green, D), 12 Aβ monomers and 36 FA anions (dark green, E). Figure S3. Propensities of helix (A), β-turn (B) and bending (C) averaged over 1000 frames (1000 ns) for of 12 Aβ monomers (grey), 12 Aβ monomers and 12 CU molecules (pink), 12 Aβ monomers and 36 CU molecules (red), 12 Aβ monomers and 12 FA anions (light green), 12 Aβ monomers and 36 FA anions (dark green). Figure S4. Lifetimes of H-bonds between Aβ peptides (left pie-charts), between Aβ and ligards (middle pie-chart) and between ligands (right pie-charts).

## References

[1] Michaels T.C.T., Šarić A., Habchi J., Chia S., Meisl G., Vendruscolo M., Dobson C.M., Knowles T.P.J. (2018). Chemical Kinetics for Bridging Molecular Mechanisms and Macroscopic Measurements of Amyloid Fibril Formation. Ann. Rev. Phys. Chem..

[2] Romero D., Aguilar C., Losick R., Kolter R. (2010). Amyloid fibers provide structural integrity to Bacillus subtilis biofilms. Proc. Natl. Acad. Sci. USA.

[3] Jacob R.S., George E., Singh P.K., Salot S., Anoop A., Jha N.N., Sen S., Maji S.K. (2016). Cell Adhesion on Amyloid Fibrils Lacking Integrin Recognition Motif. J. Biol. Chem..

[4] Matsuzaki K., Horikiri C. (1999). Interactions of amyloid beta-peptide (1-40) with ganglioside-containing membranes. Biochemistry.

[5] Zadali R., Ghareghozloo E.R., Ramezani M., Hassani V., Rafiei Y., Chiyaneh S.M., Meratan A.A. (2019). Interactions and Membrane Permeabilization of Brain Mitochondria by Amyloid Fibrils. J. Vis. Exp..

[6] Hieronymus L., Griffin S. (2015). Role of Amylin in Type 1 and Type 2 Diabetes. Diabetes Educ..

[7] Guo T., Noble W., Hanger D.P. (2017). Roles of tau protein in health and disease. Acta Neuropathol..

[8] Palop J.J., Mucke L. (2010). Amyloid-beta-induced neuronal dysfunction in Alzheimer’s disease: From synapses toward neural networks. Nat. Neurosci..

[9] Wozniak M.A., Itzhaki R.F., Shipley S.J., Dobson C.B. (2007). Herpes simplex virus infection causes cellular beta-amyloid accumulation and secretase upregulation. Neurosci. Lett..

[10] Eimer W.A., Vijaya Kumar D.K., Navalpur Shanmugam N.K., Rodriguez A.S., Mitchell T., Washicosky K.J., György B., Breakefield X.O., Tanzi R.E., Moir R.D. (2018). Alzheimer’s Disease-Associated β-Amyloid Is Rapidly Seeded by Herpesviridae to Protect against Brain Infection. Neuron.

[11] Sulzer D., Edwards R.H. (2019). The physiological role of α-synuclein and its relationship to Parkinson’s Disease. J. Neurochem..

[12] Watt B., van Niel G., Raposo G., Marks M.S. (2013). PMEL: A pigment cell-specific model for functional amyloid formation. Pigment. Cell Melanoma Res..

[13] Hardy J.A., Higgins G.A. (1992). Alzheimer’s disease: The amyloid cascade hypothesis. Science.

[14] Irwin D.J., Lee V.M., Trojanowski J.Q. (2013). Parkinson’s disease dementia: Convergence of α-synuclein, tau and amyloid-β pathologies. Nat. Rev. Neurosci..

[15] Sigurdson C.J., Bartz J.C., Glatzel M. (2019). Cellular and Molecular Mechanisms of Prion Disease. Ann. Rev. Pathol..

[16] Bharadwaj P., Wijesekara N., Liyanapathirana M., Newsholme P., Ittner L., Fraser P., Verdile G. (2017). The Link between Type 2 Diabetes and Neurodegeneration: Roles for Amyloid-β, Amylin, and Tau Proteins. J. Alzheimer’s Dis. JAD.

[17] Tumblin A., Tailor A., Hoehn G.T., Mack A.K., Mendelsohn L., Freeman L., Xu X., Remaley A.T., Munson P.J., Suffredini A.F. (2010). Apolipoprotein A-I and serum amyloid A plasma levels are biomarkers of acute painful episodes in patients with sickle cell disease. Haematologica.

[18] Ilie I.M., Caflisch A. (2019). Simulation Studies of Amyloidogenic Polypeptides and Their Aggregates. Chem. Rev..

[19] Lazo N.D., Grant M.A., Condron M.C., Rigby A.C., Teplow D.B. (2005). On the nucleation of amyloid beta-protein monomer folding. Protein Sci..

[20] Törnquist M., Michaels T., Sanagavarapu K., Yang X., Meisl G., Cohen S., Knowles T., Linse S. (2018). Secondary nucleation in amyloid formation. Chem. Commun..

[21] Eisele Y.S., Monteiro C., Fearns C., Encalada S.E., Wiseman R.L., Powers E.T., Kelly J.W. (2015). Targeting protein aggregation for the treatment of degenerative diseases. Nat. Rev. Drug Discov..

[22] Ono K., Hasegawa K., Naiki H., Yamada M. (2004). Curcumin has potent anti-amyloidogenic effects for Alzheimer’s beta-amyloid fibrils in vitro. J. Neurosci. Res..

[23] Bhat A., Mahalakshmi A.M., Ray B., Tuladhar S., Hediyal T.A., Manthiannem E., Padamati J., Chandra R., Chidambaram S.B., Sakharkar M.K. (2019). Benefits of curcumin in brain disorders. BioFactors.

[24] World Health Organization World Life Expectancy. Report 2018. https://www.worldlifeexpectancy.com/.

[25] Krishnaswamy K. (2008). Traditional Indian spices and their health significance. Asia Pac. J. Clin. Nutr..

[26] Pulido-Moran M., Moreno-Fernandez J., Ramirez-Tortosa C., Ramirez-Tortosa M. (2016). Curcumin and Health. Molecules.

[27] Kunnumakkara A.B., Bordoloi D., Padmavathi G., Monisha J., Roy N.K., Prasad S., Aggarwal B.B. (2017). Curcumin, the golden nutraceutical: Multitargeting for multiple chronic diseases. Br. J. Pharmacol..

[28] Kocaadam B., Şanlier N. (2017). Curcumin, an active component of turmeric (Curcuma longa), and its effects on health. Crit. Rev. Food Sci. Nutr..

[29] Kim Y., Clifton P. (2018). Curcumin Cardiometabolic Health and Dementia. Int. J. Environ. Res. Public Health.

[30] Di Meo F., Margarucci S., Galderisi U., Crispi S., Peluso G. (2019). Curcumin Gut Microbiota and Neuroprotection. Nutrients.

[31] Yang F.S., Lim G.P., Begum A.N., Ubeda O.J., Simmons M.R., Ambegaokar S.S., Chen P.P., Kayed R., Glabe C.G., Frautschy S.A. (2005). Curcumin Inhibits Formation of Amyloid beta Oligomers and Fibrils, Binds Plaques, and Reduces Amyloid in vivo. J. Biol. Chem..

[32] Bondi M.L., Montana G., Craparo E.F., Picone P., Capuano G., Di Carlo M., Giammona G. (2009). Ferulic Acid-Loaded Lipid Nanostructures as Drug Delivery Systems for Alzheimers Disease: Preparation, Characterization and Cytotoxicity Studies. Curr. Nanosci..

[33] Yanagisawa D., Ibrahim N.F., Taguchi H., Morikawa S., Hirao K., Shirai N., Soabe T., Tooyama I. (2015). Curcumin derivative with the substitution at C-4 position, but not curcumin, is effective against amyloid pathology in APP/PS1 mice. Neurobiol. Aging.

[34] Cox K.H., Pipingas A., Scholey A.B. (2015). Investigation of the effects of solid lipid curcumin on cognition and mood in a healthy older population. J. Psychopharmacol..

[35] Small G.W., Siddarth P., Li Z., Miller K.J., Ercoli L., Emerson N.D., Martinez J., Wong K.P., Liu J., Merrill D.A. (2018). Memory and Brain Amyloid and Tau Effects of a Bioavailable Form of Curcumin in Non-Demented Adults: A Double-Blind, Placebo-Controlled 18-Month Trial. Am. J. Geriatr Psychiatry.

[36] Zhao L.N., Chiu S.W., Benoit J., Chew L.Y., Mu Y. (2012). The effect of curcumin on the stability of Aβ dimers. J. Phys. Chem B.

[37] Awasthi M., Singh S., Pandey V.P., Dwivedi U.N. (2018). Modulation in the conformational and stability attributes of the Alzheimer’s disease associated amyloid-beta mutants and their favorable stabilization by curcumin: Molecular dynamics simulation analysis. J. Biomol. Struct. Dyn..

[38] Ngo S.T., Li M.S. (2012). Curcumin binds to Aβ1-40 peptides and fibrils stronger than ibuprofen and naproxen. J. Phys. Chem B.

[39] Kundaikar H.S., Degani M.S. (2015). Insights into the Interaction Mechanism of Ligands with Aβ42 Based on Molecular Dynamics Simulations and Mechanics: Implications of Role of Common Binding Site in Drug Design for Alzheimer’s Disease. Chem. Biol. Drug Des..

[40] Tavanti F., Pedone A., Menziani M.C. (2018). Computational Insight into the Effect of Natural Compounds on the Destabilization of Preformed Amyloid-β(1⁻40) Fibrils. Molecules.

[41] Jakubowski J.M., Orr A.A., Le D.A., Tamamis P. (2020). Interactions between Curcumin Derivatives and Amyloid-β Fibrils: Insights from Molecular Dynamics Simulations. J. Chem. Inf. Model..

[42] Bajda M., Filipek S. (2017). Computational approach for the assessment of inhibitory potency against beta-amyloid aggregation. Bioorg. Med. Chem. Lett..

[43] Ono K., Hirohata M., Yamada M. (2005). Ferulic acid destabilizes preformed β-amyloid fibrils in vitro. Biochem. Biophys. Res. Commun..

[44] Kim S., Chen J., Cheng T., Gindulyte A., He J., He S., Li Q., Shoemaker B.A., Thiessen P.A., Yu B. (2019). PubChem 2019 update: Improved access to chemical data. Nucleic Acids Res..

[45] Yanagisawa D., Shirai N., Amatsubo T., Taguchi H., Hirao K., Urushitani M., Morikawa S., Inubushi T., Kato M., Kato F. (2010). Relationship between the tautomeric structures of curcumin derivatives and their Abeta-binding activities in the context of therapies for Alzheimer’s disease. Biomaterials.

[46] Rao P.P., Mohamed T., Teckwani K., Tin G. (2015). Curcumin Binding to Beta Amyloid: A Computational Study. Chem. Biol. Drug Des..

[47] Wang J.M., Wolf R.M., Caldwell J.W., Kollman P.A., Case D.A. (2004). Development and Testing of a General Amber Force Field. J. Comput. Chem..

[48] Jakalian A., Bush B.L., Jack D.B., Bayly C.I.F. (2000). Efcient generation of high-quality atomic charges. AM1-BCC model: I. Method. J. Comput. Chem..

[49] Case D.A. (2005). Te Amber Biomolecular Simulation Programs. J. Comput. Chem..

[50] Crescenzi O., Tomaselli S., Guerrini R., Salvadori S., D’Ursi A.M., Temussi P.A., Picone D. (2002). Solution structure of the Alzheimer amyloid beta-peptide (1-42) in an apolar microenvironment. Similarity with a virus fusion domain. Eur. J. Biochem..

[51] Adelman S.A., Doll J.D. (1974). Generalized Langevin equation approach for atom/solid-surface scattering: Collinear atom/harmonic chain model. J. Chem. Phys..

[52] Berendsen H.J.C., Postma J.P.M., van Gunsteren W.F., DiNola A., Haak J.R. (1984). Molecular dynamics with coupling to an external bath. J. Chem. Phys..

[53] Maier J.A. (2015). f14SB: Improving the Accuracy of Protein Side Chain and Backbone Parameters from f99SB. J. Chem. Teory Comput..

[54] Darden T., York D., Pedersen L. (1993). Particle mesh Ewald: An N-log(N) method for Ewald sums in large systems. J. Chem Phys..

[55] Ciccotti G., Ryckaert J.P. (1986). Molecular dynamics simulation of rigid molecules. Comput. Phys. Rep..

[56] Case D.A., Ben-Shalom I.Y., Brozell S.R., Cerutti D.S., Cheatham T.E., Cruzeiro V.W.D., Darden T.A., Duke R.E., Ghoreishi D., Gilson M.K. (2018). AMBER.

[57] Roe D.R., Cheatham T.E. (2013). PTRAJ and CPPTRAJ: Sofware for Processing and Analysis of Molecular Dynamics Trajectory Data. J. Chem. Teory Comput..

[58] Mishra S., Palanivelu K. (2008). The effect of curcumin (turmeric) on Alzheimer’s disease: An overview. Ann. Indian Acad. Neurol..

[59] Pawar Y.B., Munjal B., Arora S., Karwa M., Kohli G., Paliwal J.K., Bansal A.K. (2012). Bioavailability of a lipidic formulation of curcumin in healthy human volunteers. Pharmaceutics.

[60] Lao C.D., Ruffin M.T., Normolle D., Heath D.D., Murray S.I., Bailey J.M., Boggs M.E., Crowell J., Rock C.L., Brenner D.E. (2006). Dose escalation of a curcuminoid formulation. BMC Complement. Altern. Med..

[61] Mattila P., Pihlava J.M., Hellstro¨m J. (2005). Contents of phenolic acids, alkyl- and alkenylresorcinols, and avenanthramides in commercial grain products. J. Agricult. Food Chem..

[62] Moore J., Hao Z., Zhou K., Luther M., Costa J., Yu L. (2005). Carotenoid, tocopherol, phenolic acid, and antioxidant properties of Maryland-grown soft wheat. J. Agricult. Food Chem..

[63] Graf E. (1992). Antioxidant potential of ferulic acid. Free Rad. Biol. Med..

[64] Mateo Anson N., Berg v.d.R., Havenaar R., Bast A., Haenen G.R.M.M. (2008). Ferulic acid from aleurone determines the antioxidant potency of wheat grain (Triticum aestivum L.). J. Agricult. Food Chem..

[65] Ou S., Kwok K.C. (2004). Ferulic acid: Pharmaceutical functions, preparation and applications in foods. J. Sci. Food Agric..

[66] Srinivasan M., Sudheer A.R., Menon V.P. (2007). Ferulic acid: Therapeutic potential through its antioxidant property. J. Clin. Biochem. Nutr..

[67] Barone E., Calabrese V., Mancuso C. (2009). Ferulic acid and its therapeutic potential as a hormetin for age-related diseases. Biogerontology.

[68] Zhao Z., Moghadasian M.H. (2008). Chemistry, natural sources, dietary intake and pharmacokinetic properties of ferulic acid: A review. Food Chem..

[69] Wu K., Wang Z.Z., Liu D., Qi X.R. (2014). Pharmacokinetics, brain distribution, release and blood-brain barrier transport of Shunaoxin pills. J. Ethnopharmacol..

[70] Reinke A.A., Gestwicki J.E. (2007). Structure-activity relationships of amyloid beta-aggregation inhibitors based on curcumin: Influence of linker length and flexibility. Chem. Biol. Drug Des..

[71] Wishart D.S., Feunang Y.D., Guo A.C., Lo E.J., Marcu A., Grant J.R., Sajed T., Johnson D., Li C., Sayeeda Z. (2018). DrugBank 5.0: A major update to the DrugBank database for 2018. Nucleic Acids Res..

[72] Modi M.A., Kale N.V., Patel J.H., Varia R.D., Modi F.D., Vihol P.D. (2019). Pharmacokinetics of ferulic acid following oral administration ethyl ferulate alone and in combination with piperine in rats. Ann. Phytomed..

[73] Balchin D., Hayer-Hartl M., Hartl F.U. (2016). In vivo aspects of protein folding and quality control. Science.

[74] Vitalis A., Caflisch A. (2010). Micelle-like architecture of the monomer ensemble of Alzheimer’s amyloid-β peptide in aqueous solution and its implications for Aβ aggregation. J. Mol. Biol..

[75] Roche J., Shen Y., Lee J.H., Ying J., Bax A. (2016). Monomeric Aβ1–40 and Aβ1–42 Peptides in Solution Adopt Very Similar Ramachandran Map Distributions That Closely Resemble Random Coil. Biochemistry.

