# Supplementary material for: Curcumin Inhibits the Primary Nucleation of Amyloid-Beta Peptide: A Molecular Dynamics Study
Source: Biomolecules. 2020 Sep 15;10(9):1323. doi: 10.3390/biom10091323 (PMC7563689; doi:10.3390/biom10091323)
Supplement: Supplementary file 1 [file biomolecules-10-01323-s001.zip › biomolecules-907369-supporting materials/Figure_S2.docx]

A

B

C

D

E

**Figure S2.** RMSDs (˗˗˗ single run, ---- average of 3 runs) averaged over 50 frames (50 ns) for 12 Aβ monomers (grey, A), 12 Aβ monomers and 12 CU molecules (pink, B), 12 Aβ monomers and 36 CU molecules (red, C), 12 Aβ monomers and 12 FA anions (light green, D), 12 Aβ monomers and 36 FA anions (dark green, E).
