# Supplementary material for: Curcumin Inhibits the Primary Nucleation of Amyloid-Beta Peptide: A Molecular Dynamics Study
Source: Biomolecules. 2020 Sep 15;10(9):1323. doi: 10.3390/biom10091323 (PMC7563689; doi:10.3390/biom10091323)
Supplement: Supplementary file 1 [file biomolecules-10-01323-s001.zip › biomolecules-907369-supporting materials/Figure_S3.docx]

A

B

C

**Figure S3.** Propensities of helix (A), β-turn (B) and bending (C) averaged over 1000 frames (1000 ns) for of 12 Aβ monomers (grey), 12 Aβ monomers and 12 CU molecules (pink), 12 Aβ monomers and 36 CU molecules (red), 12 Aβ monomers and 12 FA anions (light green), 12 Aβ monomers and 36 FA anions (dark green).
