# Supplementary material for: Curcumin Inhibits the Primary Nucleation of Amyloid-Beta Peptide: A Molecular Dynamics Study
Source: Biomolecules. 2020 Sep 15;10(9):1323. doi: 10.3390/biom10091323 (PMC7563689; doi:10.3390/biom10091323)
Supplement: Supplementary file 1 [file biomolecules-10-01323-s001.zip › biomolecules-907369-supporting materials/Figure_S4.docx]

**Figure S4.** Lifetimes of H-bonds between Aβ peptides (left pie-charts), between Aβ and ligards (middle pie-chart) and between ligands (right pie-charts).
